# Association of Atrial Fibrillation and Cognitive Dysfunction: A Comprehensive Narrative Review of Current Understanding and Recent Updates

**DOI:** 10.3390/jcm13185581

**Published:** 2024-09-20

**Authors:** Siddhant Passey, Jay Patel, Haris Patail, Wilbert Aronow

**Affiliations:** 1 Department of Internal Medicine, University of Connecticut School of Medicine, Farmington, CT 06030, USA; passey@uchc.edu (S.P.); jaypatel1@uchc.edu (J.P.); 2 Department of Cardiology, Westchester Medical Center New York Medical College, Valhalla, NY 10595, USA; haris.patail@wmchealth.org; 3 Department of Medicine, Westchester Medical Center New York Medical College, Valhalla, NY 10595, USA

**Keywords:** atrial fibrillation, cognitive dysfunction, dementia, neuroimaging, cerebral infarcts, anticoagulation, catheter ablation

## Abstract

Atrial fibrillation (AF) is the most common sustained arrhythmia in adults. The prevalence of both AF and dementia is steadily rising and is expected to rise further in the coming decades. There is increasing evidence to suggest an association between AF and various degrees of cognitive dysfunction, from mild cognitive impairment to severe dementia. In this review, we aimed to discuss the epidemiological aspects, pathophysiological mechanisms, role of neuroimaging, impact of treatment modalities, and clinical and socioeconomic impact of this association. Numerous observational studies and meta-analyses have revealed this association to exist in AF patients with and without a history of stroke, and the association also persists after adjusting for shared risk factors such as hypertension and diabetes mellitus. Various pathophysiological mechanisms have been proposed for this association, including silent cerebral infarcts, cerebral microbleeds, cerebral hypoperfusion, inflammation, and atherosclerosis. While neuroimaging findings have been utilized to suggest some of these pathophysiological mechanisms, more studies are needed to further elucidate this and to determine the potential role of neuroimaging in altering anticoagulation and other treatment decisions. Anticoagulants have shown effectiveness in reducing the rate of cognitive decline in AF patients; however, their role in low-risk AF patients remains under investigation. Even though AF patients receiving catheter ablation may have post-operative cognitive dysfunction in the short term, long-term follow-up studies have shown an improvement in cognitive function following ablation. Cognitive decline in AF patients often occurs with greater functional decline and other psychosocial impairments such as depression and anxiety and future research on this association must incorporate aspects of social determinants of health and associated outcomes.

## 1. Introduction

Atrial fibrillation (AF) is the leading form of sustained arrhythmia in adults [1], with a 2019 prevalence of more than 59 million individuals worldwide [2]. The prevalence has been steadily rising from 33.5 million in 2010 and is projected to rise further in the coming decades. The number of individuals with dementia had an estimated rise from 20.3 million in 1990 [3] to 57.4 million in 2019 worldwide [4]. Future estimates predict that by 2050, the prevalence of dementia will increase to 152.8 million cases globally [4].

In the last two decades, many studies have described a relationship between AF and cognitive decline (Table 1). However, significant knowledge gaps remain, and it is still uncertain if this association is causal. Understanding the pathophysiology of this association and exploring different management strategies could improve the quality of life of individuals with AF and decrease the associated financial burden.

In this review, we aimed to (a) explore the epidemiological aspects of the association between AF and cognitive impairment, including the role of history of stroke, age, and gender; (b) review the proposed pathophysiological mechanisms implicated in this association such as silent cerebral infarcts, cerebral microbleeds, and cerebral hypoperfusion; (c) discuss the role of neuroimaging as it influences a variety of treatment and management strategies for AF and its association with cognitive function; (d) review the impact of different treatment modalities for AF on cognitive function; and (e) explore the clinical and socioeconomic impact of this association.

## 2. Association between Atrial Fibrillation and Cognitive Impairment

The first study to describe a relationship between AF and cognitive impairment was the Rotterdam study which showed that the patients with AF had double the propensity to have co-existing dementia when compared to patients without a history of AF [5]. Since then, numerous studies have been conducted on this association. Individual studies on this relationship encompassing a variety of study designs have been inconsistent in their conclusions (Table 1). Various factors have been attributed to this inconsistency. One factor is the heterogeneity in diagnosing and defining cognitive impairment in clinical practice. Different assessment methods, the majority of which are highly subjective, have been used in studies, assessing varying degrees of cognitive impairment. While some of these methods, such as the International Classification of Diseases (ICD) or Diagnostic and Statistical Manual of Mental Disorders (DSM) criteria utilizing clinical assessment for diagnosis by a physician have a propensity for underdiagnosis [6], alternative screening methods such as neuropsychometric evaluation or singular screening approaches may result in either excessive diagnosis or inadequate detection of cognitive impairment. Moreover, it has been shown that the different prevailing diagnostic criteria for dementia can exhibit significant variance, resulting in a ten-fold difference in the number of individuals classified as affected [7]. AF and cognitive impairment share a complex relationship due to numerous common risk factors such as advancing age, diabetes, hypertension, coronary artery disease, dyslipidemia, sleep apnea, chronic kidney disease, heart failure, lack of physical activity, obesity, and increased alcohol use. However, some studies have shown the persistence of association between these two entities despite adjusting the common risk factor data metrics [8,9,10]. Lastly, a majority of these studies have been done in Western populations; therefore, the strength of this association in non-Western populations has not been defined.

### 2.1. Impact of History of Stroke on Association between Atrial Fibrillation and Cognitive Impairment

AF causes a five-time rise in the risk of cerebral ischemic events, accounting for >15% of strokes in the United States [11]. It is also recognized that stroke is a common cause of dementia, with a meta-analysis performed on new stroke patients demonstrating that 10% of the study subjects develop dementia after the stroke [12]. There is considerable scientific literature linking AF with dementia among individuals who have a previous history of stroke [13,14,15]. A meta-analysis of seven studies showed that the risk of developing dementia after stroke was more than double in patients who had a history of AF compared to those who did not have a history of AF [16].

An association between cognitive impairment and AF in patients who did not have a history of stroke has also been demonstrated in more recent studies, irrespective of shared risk factors (Table 1). While some studies have found a link between these two entities in patients without stroke history [5,17,18], other studies have not found such an association [19,20,21]. Three independent meta-analyses by Papanastasiou et al. [22], Kalantarian et al. [23], and Santangeli et al. [24] have shown a statistically significant association between AF and dementia in individuals who had not previously experienced a stroke. The discrepancy in the results of different observational studies could be attributed to the difference in power and study design as well as the different outcome measures used to quantify cognitive impairment.

### 2.2. Role of Age and Gender in Association between Atrial Fibrillation and Cognitive Impairment

Some studies have shown an interesting role of the age of individuals in the association between AF and cognitive impairment. In a prospective cohort study, the odds ratio for association of nonspecific dementia for subjects < 70 years old was 2.87 (*p* < 0.0001), compared to an odds ratio of 0.96 (*p* = 0.077) for individuals of 80–89 years of age [17]. In another prospective cohort study, which was an update to the Rotterdam study, the authors found that the association between incident AF and risk of dementia was statistically significant in the younger cohort (<67 years) compared to the older cohort (>67 years).

Some studies have also pointed to gender differences in the link between AF and cognitive impairment. A subgroup analysis of the Rotterdam study [5] showed that the association of AF individually with both cognitive impairment and dementia was statistically significant only for women and not for men. In a prospective cohort study by Roberts et al., AF showed an elevated risk of non-amnestic mild cognitive impairment (MCI) only in women and not in men [25]. However, in an analysis of postmenopausal women between the ages of 65 to 79 who were part of the Women’s Health Initiative Memory Study, AF did not have a significant association with either mild cognitive impairment or probable dementia [20].

### 2.3. Type of AF and Association with Cognitive Dysfunction

AF has been classified according to its duration and length of episodes, and the classification has changed over the years [26]. Based on current data, it is unclear whether there is a dose–response relationship between the AF burden and degree of cognitive dysfunction. A study conducted on 325 participants of the ARIC study compared persistent (AF burden 100%) and paroxysmal AF (AF burden 1–6%), based on ECG patch monitoring that the participants wore for 14 weeks [27]. The study compared a battery of neurocognitive assessments and concluded that only persistent AF and not paroxysmal AF was independently associated with cognitive decline. Another cross-sectional study of 269 participants was completed in Italy and investigated changes in cognitive function based on whether participants were in sinus rhythm, paroxysmal AF (≥1 episode of AF lasting < 48 h), or chronic AF (arrhythmia lasting > 6 months) [28]. They used MMSE scores and scores of less than 24 were classified as cognitively impaired. The study showed a 3.3-fold increase in the incidence of cognitive impairment in the chronic AF cohort which was statistically significant compared to the sinus rhythm and a 1.2-fold increase in cognitive impairment in the paroxysmal AF group when compared to the normal sinus rhythm group, which was not statistically significant. While these studies suggest a dose–response relationship between AF burden and cognitive impairment, they were small studies, and more research is needed on this topic.

**Table 1:** Major studies describing an association between atrial fibrillation and cognitive dysfunction. Over the last 3 decades, a variety of study designs have been utilized to assess for an association between AF and cognitive impairment. Studies have also used different outcomes to assess cognitive impairment.

Study (First Author, Year)	Study Design	Number of Subjects, % Male	Age (Years)	Follow-Up Years	Outcome Assessed	Results
Ott, 1997 [5]	Cross-sectional Rotterdam study	6584, 40.8	Mean 69.2 (55–106)	0 (Cross sectional design)	Cognitive impairment defined as MMSE score < 26	Association between AF and dementia (OR 2.3, 95% CI 1.4 to 3.7) and AF and cognitive function (OR 1.7, 95% CI 1.2 to 2.5).
Tilvis, 2004 [29]	Prospective cohort	650, 60	≥75 at time of enrollment	5 and 10	Decrease in MMSE by 4 points or increase in Clinical Dementia Rating class	At 5 years, AF associated with cognitive decline RR 2.9 (1.3–6.1)
Elias, 2006 [30]	Prospective cohort, including only men	1011, 100	Median 61 (37–89)	0.75	Cognitive function score—Framingham neuropsychological review panel	AF associated with Global Composite score below 25th percentile on OR 4.10 (1.84–8.74)
Forti, 2007 [31]	Retrospective cohort	611, 37	75.2(±9.0)	Subjects with MCI: 3 Subjects with normal cognition: 4	MMSE and neuropsychological assessment battery	MCI group: AF associated with dementia, HR 4.63 (1.72–12.46) Normal cognition: AF not associated with dementia, HR 1.10 (0.40–2.98)
Peters, 2009 [21]	Prospective cohort, including only individuals with hypertension	3336, 39.6	≥80	2	DSM-IV criteria for dementia, MMSE < 24 or annual decline ≥ 3 for cognitive decline	No significant association between AF and dementia (multivariate HR 1.031, 95% CI 0.619–1.718) or between AF and cognitive decline (multivariate HR 1.080, 95% CI 0.798–1.463)
Bunch, 2010 [17]	Prospective cohort	37,025, 60.6	60.6(±17.9)	5	ICD-9 code for dementia	Significant association between AF and nonspecific dementia (OR 1.44 *p* < 0.0001)
Dublin, 2011 [32]	Prospective cohort	3045, 40	74.3 (70.3–79.5)	6.8	Neuropsychological and neurological assessment and DSM-IV criteria for dementia	Significant association between AF and dementia (HR 1.38, 95% CI 1.10 to 1.73)
Marengoni, 2011 [19]	Retrospective cohort	63,185, NR	≥75	6	DSM-III Criteria for dementia	No significant association identified between AF and incident dementia (HR 0.9, 95% CI 0.7–1.7)
Marzona, 2012 [9]	Post hoc analysis of two randomized control trials	31,560, 70	67 ± 7	4.7	New dementia; MMSE < 24, cognitive decline: ≥3 point decline in MSME	Statistically significant association between AF and cognitive decline (HR 1.14, 95% CI 1.036 to 1.26), and between AF and new dementia (HR 1.30, 95% CI 1.14 to 1.49)
Haring, 2013 [20]	Prospective cohort study, including only women	6445, 0	65–79	8.4	MCI or PD via 3MSE score; neurocognitive and neuropsychiatric testing	Association not identified between AF and MCI or PD (*p* value = 0.2493)
Thacker 2013 [33]	Prospective cohort study	5888, 41	73.0 ± 5.4	7	3MSE score	Statistically significant faster decline in 3MSE score after incident AF compared with no prior AF
Chen, 2014 [34]	Prospective cohort	935, 38	62 ± 4	Median 10.6	Battery of tests for cognitive assessment	AF associated with cognitive decline in stroke-free individuals who had evidence of subclinical infarcts
De Bruijn, 2015 [35]	Prospective cohort study embedded within Rotterdam study	6514, 41	Patients with AF: 75.7 (±8.1); patients without AF: 68.3 (±8.5)	20	Dementia diagnosis determined by a consensus panel using DSM-III criteria	Prevalent AF associated with increased risk of dementia (HR 1.33, 95% CI 1.02–1.73). Incident AF associated with increased risk of dementia in younger (<67 years) participants (1.81, 95% CI 1.11–2.94) compared to older participants (>67 years) (1.12, 95% CI 0.85–1.46)
Chen, 2018 [8]	Prospective cohort	12,515, 44	56.9 ± 5.7	20	Dementia assessed by algorithm based on DSM-V and National Institute on Aging—Alzheimer’s Association work groups	Significant association between AF and risk of dementia (HR 1.23, 95% CI 1.04 to 1.45) after adjustment for cardiovascular risk factors including ischemic stroke
Nishtala, 2018 [36]	Cross-sectional and longitudinal analysis of Framingham heart study including original and offspring cohorts	2682, 45	72 ± 9	Mean follow-up 3 years for original cohort and 6 years for offspring cohort	Multiple neuropsychological tests covering a variety of cognitive domains	Both prevalent and interim AF associated with longitudinal decline in executive function
Kim, 2019 [18]	Prospective cohort	262,611, 44.2	>60	1–8	Korean Dementia Screening Questionnaire (KDSQ)	Risk of dementia significantly increased by incident AF, HR 1.52 (95% CI 1.43–1.63), compared to AF-free individuals; risk similarly increased after adjusting for stroke (HR 1.27, 95% CI 1.18–1.37)
Bailey, 2021 [10]	Prospective cohort	25,980, 44.5	64 ± 9.3	8.06	Six-item screening including MoCA	At baseline, AF associated with lower cognitive performance; incident AF also associated with cognitive impairment

AF—atrial fibrillation, MCI—mild cognitive impairment, PD—Probable Dementia, MMSE—Mini-Mental Status Exam, 3MSE—Modified Mini-Mental State Examination, MoCA—Montreal Cognitive Assessment, ICD—International Classification of Diseases, DSM—Diagnostic and Statistical Manual of Mental Disorders, OR—odds ratio, HR—hazard ratio, RR—Relative Risk, CI—confidence interval, NR—not reported.

## 3. Proposed Pathophysiologic Mechanisms

**Figure 1:**
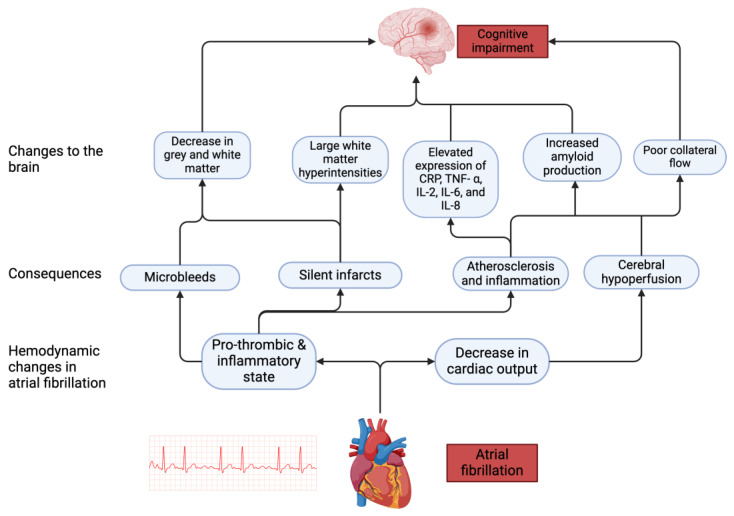
Pathophysiological mechanisms associated with cognitive impairment in AF. AF leads to hemodynamic changes which in turn cause downstream effects leading to changes in the brain that impair cognitive function.

Over the years, various theories have been suggested to explain the relationship that is shared between AF and cognitive function. These include shared risk factors, silent cerebral infarcts, cerebral microbleeds, cerebral hypoperfusion, inflammation, and atherosclerosis. (Figure 1)

### 3.1. Silent Cerebral Infarcts

One primary proposed mechanism for the association between AF and cognitive decline is silent cerebral infarcts. “Silent” pertains to ischemic brain lesions that lack corresponding clinical syndromes as determined through history or clinical examination [37]. Most silent infarcts are found in the subcortical white matter, while 10% are in the cortical region. The prevalence of brain infarcts in AF ranges between 15 to 50% [38]. In a meta-analysis by Kalantarian et al., it was observed that silent infarcts were increased as much as 2.6-fold in patients with AF compared to those without AF [23]. A meta-analysis completed by Azeem et al. evaluated 30 studies focusing on cognitive impairment and silent brain infarcts within the general population [39]. Their analysis found that most studies noted an increased risk of worse cognition and development of dementia in patients with silent cerebral infarcts. Additionally, in a prospective study completed by Kuhne et al., AF patients who were anticoagulated underwent magnetic resonance imaging (MRI) scanning to obtain baseline findings and repeated the MRI scan after two years. This study demonstrated that 5.5% of patients with AF had a new brain infarct on MRI [40]. This further shows that despite anticoagulation, these patients had a high incidence of new brain infarcts, which can negatively impact cognition over time. A study completed by Ryden et al. also demonstrated similar findings regarding the impact of AF on the development of silent infarcts [41]; however, it showed that AF was associated with an increase in symptomatic strokes and MRI findings of large infarcts.

### 3.2. Cerebral Microbleeds

Cerebral microbleeds are characterized by their small size, typically 2–5 mm in diameter and their ovoid and spherical shape, and they appear as a profound hypointense signal on T2 weighted imaging [42]. A retrospective analysis found that AF patients had a higher prevalence of cerebral microbleeds (30.5%) compared to a cohort without AF (22.8%) [43]. Another recent study confirms similar prevalence rates of cerebral microbleeds in patients with AF to be as high as 28.3% [44]. A study by Akoudad et al. showed that populations with more than four microbleeds detected on MRI had worse scores on the letter digit substitution test, Stroop reading, naming subtasks, immediate 15-word learning tests, and Purdue pegboard test [45]. Additionally, this study noted that infratentorial microbleeds lead to poorer motor speed performance, while lobar microbleeds lead to decreased executive function and information processing speed and poorer memory. The increased incidence of microbleeds seen in AF populations can have detrimental impacts on cognition.

### 3.3. Cerebral Hypoperfusion

Another proposed mechanism for cognitive dysfunction in AF patients is cerebral hypoperfusion. A prospective study conducted by Gardarsdottir et al. utilized phase contrast MRI to estimate cerebral blood flow and perfusion in three patient cohorts [46]. They observed that the cohort with persistent AF had the lowest flow rate at 472.1 mL/min followed by the paroxysmal AF cohort and no AF cohort that had rates of 512.3 mL/min and 541.0 mL/min, respectively. A possible rationale for this decrease in perfusion could be attributed to the increase in beat-to-beat variation, which is a common phenomenon seen in AF. Given the brain’s high metabolic activity and limited intracellular energy stores, there is a large dependence on cerebral blood flow which is possibly impacted in patients with AF. Therefore, cerebral hypoperfusion observed in AF can lead to significant detrimental impacts on cognition over time.

Chronically decreased blood flow has been described as an important pathological event in the development of Alzheimer’s disease. Cerebral hypoperfusion could play a role in advancing cognitive decline by either triggering the amyloid cascade or being induced by and intensifying amyloid beta production [47]. This link between cerebral hypoperfusion and Alzheimer’s disease explains the higher rate of cognitive decline in AF patients. This predilection is an important consideration that has yet to be fully explored.

### 3.4. Inflammation and Atherosclerosis

Based on expert opinion, AF patients face an increased likelihood of developing atherosclerosis. [48]. Additionally, it has been previously established that certain markers associated with inflammation, namely C-reactive protein, tumor necrosis factor-α, interleukins -2, 6, and 8, are often elevated in AF [49]. The combination of inflammation and atherosclerosis may point to a way that cognition can be affected. More large-scale clinical trials are needed to directly explore these effects as the current literature has not directly explored how these elevated biomarkers commonly observed in AF can lead to long-term cognitive impacts.

## 4. Role of Neuroimaging

Individuals diagnosed with AF frequently exhibit brain damage that goes undetected during clinical evaluation. In a comprehensive study involving over 1000 patients with atrial-fibrillation-related vascular events, it was discovered that 24.6% of them had pre-existing cognitive impairment along with specific neuroimaging findings. Moreover, patients with pre-existing cognitive impairment experienced poorer functional outcomes after two years, regardless of the presence or absence of stroke [50]. Neuroimaging has significantly advanced our comprehension of the pathways through which AF may induce cognitive dysfunction. Cranial MRI and computed tomography (CT) are the most commonly utilized neuroimaging modalities. However, due to their limited ability to detect prior ischemic events, CT scans have largely been supplanted by MRI for such studies [51]. Brain MRI can identify patterns in otherwise asymptomatic individuals such as silent cerebral infarction, white matter hyperintensities, brain volume, and cerebral microbleeds, which are pivotal in understanding the hypothesized mechanism of association between AF and cognitive impairment [45,52,53]. (Figure 1) Although, at present, the clinical implications of these observations remain largely unexplored, we present an overview of the typical neuroimaging findings in patients with AF, along with studies that have contributed to our current comprehension of their role in cognitive impairment in AF patients (Table 2).

### 4.1. Silent Cerebral Infarcts

The tendency of AF patients to develop intracardiac thrombus leading to potential cerebral embolization is well established. It is then conceivable that subclinical microstructural brain parenchymal damage may be present in this population. Supporting this conjecture are the studies employing systematic neuroimaging, which reveal that silent cerebral infarcts are detected in up to five times as many cases at the time of AF diagnosis compared to symptomatic infarctions [54]. Notably, AF correlates with silent cerebral infarcts even in individuals lacking a history of clinically apparent stroke [23]. It was also noted that both clinically evident infarcts and silent cerebral infarcts had comparable effects on cognitive decline [40]. The prevalence of silent cerebral infarcts exhibited similarity between cohorts with paroxysmal and persistent AF; however, individuals with persistent AF demonstrated a higher lesion burden per capita [53]. Thus, the risk of developing silent-cerebral-infarct-related dementia in patients with AF appears to persist independent of symptomatology and AF duration.

### 4.2. Brain Volume

Individuals diagnosed with AF, even in the absence of cerebral infarctions, exhibit lesser brain volume relative to controls. Moreover, AF is linked to a decline in total cerebral gray matter and is more pronounced among patients with AF diagnosed earlier, particularly those presenting with a persistent pattern of this arrhythmia [55]. The Atherosclerosis Risk in Communities (ARIC) Neurocognitive Study demonstrated that AF was associated with smaller brain volumes, including reduced gray matter and hippocampal volumes [52]. However, in a cross-sectional study, Knecht et al. identified hippocampal atrophy without any difference in other radiographic measures in AF patients with a negative history of stroke, and this finding also correlated with memory impairments [56]. Interestingly, in a population-based study by Graff-Radford, J et al., AF was linked to a decreased volume of total gray matter but showed no evidence of alterations in hippocampal volume [57].

Thus, the true nature of the association between low brain volume and cognitive decline in AF is still conflicting. While observational studies point towards the association of brain volume alterations with the deterioration of cognitive abilities in AF, there is emerging evidence suggesting that the causal effect of AF on decreased gray matter volume might be mediated by ischemic stroke [58]. Therefore, in the current scenario, it is logical for the scientific community to direct efforts towards gathering concrete evidence establishing either the concomitant or the causal relationship between the two [59].

### 4.3. Cerebral Microbleeds

Although microbleeds have been associated with poor cognitive performance, suggesting they may contribute to cognitive decline in AF [60], there currently remains a lacuna in research investigating microbleeds and cognitive function in AF patients without stroke. In a retrospective study conducted by Song T.J. et al. that included 507 patients with AF and ischemic stroke, cerebral microbleeds were observed in 30% of the patients. After a follow-up period of more than two years, it was concluded that the burden of microbleeds correlated with mortality associated with ischemic stroke. Additionally, a higher risk of future intracerebral hemorrhage was associated with the presence of lobar microbleeds [61]. The underlying physiological explanation behind the association of cerebral microbleeds and cognitive impairment is not well established. One of the proposed theories suggests that cerebral microbleeds disrupt critical tracts in the white matter or cortical regions, leading to cognitive dysfunction [62].

**Table 2:** Major studies conducted on neuroimaging findings seen in patients with atrial fibrillation and their association with cognitive dysfunction. A variety of study designs have been utilized to assess for neuroimaging findings including silent cerebral infarcts, brain volume, and cerebral microbleeds.

Silent Cerebral Infarcts						
First Author, Year	Study Design	No. of Subjects	Age (Years)	Follow-Up Period	Outcomes Assessed	Results
Kalantarian, 2014 [23]	Systematic review and meta analysis	5317	Mean age 50–83.6	37 months-26 years	Prevalence of SCI in stroke-free patients with AF	AF was associated with SCI in patients without any history of stroke (OR 2.6, 95% CI 1.8–3.8)
Gaita, 2013 [53]	Cross-sectional	270	Paroxysmal AF: 58.6 ± 10.2; persistent AF: 61.2 ± 10.9 Controls: 59.7 ± 13.1	NR	a. Prevalence of SCI in AF patients b. RBANS score	a. SCI prevalence was 89% in paroxysmal AF, 92% in persistent AF, 46% in controls (*p* < 0.01); b. RBANS score in persistent AF was 82.9 ± 11.5, vs. 86.2 ± 13.8 in paroxysmal, vs. 92.4 ± 15.4 points in controls (*p* < 0.01)
Kuhne, 2022 [40]	Prospective cohort (Swiss AF)	1227	71.4 ± 8.4	24 months	a. Incidence of SNCI/LNCCI; b. Impact of brain infarcts on Cognitive Construct score	a. New lesion was discovered in 68 patients (5.5%). b. Change in Cognitive Construct score was −0.12 (95% CI −0.22; −0.07) in patients with new infarct vs. 0.07 (95% CI −0.09; 0.25) in those without
Marfella, 2013 [63]	Observational	704	NR	37.5 ± 1.6 months	Prevalence of SCI in diabetic patients with silent AF episodes	Greater prevalence of SCI in diabetics with silent AF when compared to healthy controls (61% vs. 29%; *p* < 0.01)
Chen, 2014 [34]	Prospective cohort (ARIC)	935	61.5 ± 4.3	20 years	Annual Digital Symbol Substitution and word fluency decline rate in AF patients with SCI	In AF patients with SCI average annual rate of Digital Symbol Substitution decline was −1.51 (95% CI −3.02 to −0.01; *p* = 0.049) and word fluency decline was −2.65 (95% CI 4.26 to −1.03; *p* = 0.002)
**Brain volume**						
Knecht, 2008 [56]	Cross-sectional	533	AF patients: 60 ± 12 Controls: 64 ± 7	NR	Radiographic cranial volume assessment in patients with AF	AF is independently associated with hippocampal atrophy (regression coefficient −0.272, SE 0.129; *p* < 0.01)
Banerjee, 2020 [50]	Prospective observational (CROMIS-2)	1102	76 ± 10.1	2 years	Radiographic cranial volume assessment in AF patients with pre-existing impairment in cognitive functions	Medial temporal atrophy was independently associated with pre-existing cognitive impairment in AF patients (OR 1.61; 95% CI, 1.34 to 1.95; *p* < 0.0001)
Stefansdottir, 2013 [55]	Cross-sectional analysis	4251	76 ± 5	7.6 ± 7 years	Radiographic cranial volume assessment in AF patients	a. Total brain volume was 1043.7 mL (95% CI 1029.1–1058.3) in AF patients vs. 1059.0 mL (95% CI 1045.8–1072.3) in controls; *p* < 0.01.b. Gray matter was 645.2 mL (95% CI 636.1–654.4) in AF patients vs. 657.6 mL (95% CI 649.3–665.8) in controls; *p* < 0.001. c. White matter volume was 376.5 mL (95% CI 369.5–383.5) in AF patients vs. 381.4 mL (95% CI 375.0–387.7) in controls; *p* < 0.05.
**Cerebral microbleeds**						
Song, T. J (2014) [61]	Retrospective, observational study	504	70 ± 11	2.5 years	a. CMB prevalance in ischemic stroke patients with nonvalvular AF; b. mortality with CMBs vs. without CMBs in patients with stroke and nonvalvular AF	a. CMB prevalence 30.7% in ischemic stroke patients with nonvalvular AF. b. Patients with CMBs had greater mortality compared to those without (41.9% vs. 31.8%, *p* = 0.028) in patients with stroke and nonvalvular AF
Conen, 2019 [64]	Prospective cohort study	1737	73 ± 8	NR	Decline in MoCA score in AF patients with CMBs	The decline in MoCA score in AF patients having CMBs was not statistically significant 0.08 (−0.03 to 0.19; *p* = 0.16)
Akoudad, 2016 [45]	Prospective population-based study	4841	59.6 ± 7.8	4.8 years	Association of CMBs with risk for dementia	CMBs were associated with a greater risk of developing dementia and cognitive impairment (HR, 2.02; 95% CI, 1.25–3.24)

AF—atrial fibrillation, NR—not reported, SCI—silent cerebral infarcts, SNCI—small non-cortical infarcts, LNCCI—large non-cortical or cortical infarcts, CMBs—cerebral microbleeds, MoCA—Montreal Cognitive Assessment, RBANS—Repeatable Battery for the Assessment of Neuropsychological Status, OR—odds ratio, HR—hazard ratio, CI—confidence interval, SE—standard error.

### 4.4. How Do Neuroimaging Findings in Atrial Fibrillation Impact Its Management?

The relevance of silent cerebral infarcts for adjusting the CHA_2_DS_2_-VASc score (which is used to estimate thromboembolic risk in patients with AF [65]) and initiating prophylactic anticoagulant therapy in those otherwise belonging to the low-risk category remains uncertain [38]. Despite varied practice approaches among neurologists, many advocate for oral anticoagulant therapy in cases of silent cerebral infarcts presumed to be due to an embolus of cardiac origin. An intracranial hemorrhage warrants withholding anticoagulation, but cerebral microbleeds, which increase the risk of developing intracranial hemorrhage, do not. AF patients are often prescribed anticoagulation therapy, which increases their risk of developing cerebral microbleeds. Concurrent cerebral microbleeds with an acute stroke elevate the risk of recurrent ischemic stroke [66]. In a small prospective MRI study comprising 69 subjects, it was observed that individuals with AF who used warfarin developed new cerebral microbleeds after one year. However, there was no association found between the use of direct oral anticoagulants (DOACs) or antiplatelet agents and the development of cerebral microbleeds [67]. In a recent case-control study, a robust association was found between the presence of ≥10 MRI-proven cerebral microbleeds and the subsequent occurrence of intracerebral hemorrhage in patients with AF. Notably, lobar brain regions were consistently implicated whenever there were ≥10 cerebral microbleeds. These findings suggest that brain MRIs could assist in optimizing anticoagulation decisions for certain AF patients [68].

### 4.5. Should Routine Neuroimaging Screening and Follow-Up Imaging Be Recommended in All Atrial Fibrillation Patients?

Despite its promise, there is a paucity of robust guidelines on the significance of neuroimaging in cases of AF in the absence of newly developed or exacerbated cognitive impairment. The early identification of cognitive deterioration via regular cognitive assessments in AF patients should be followed with imaging if interventions for dementia prevention in AF are developed. However, in the absence of such interventions, the rationale of routine imaging in AF patients remains unjustified [66]. There is increasing evidence suggesting that AF is associated with an augmented propensity for alterations in brain imaging over extended durations. In a recent longitudinal investigation carried out over 11 years, the AF cohort exhibited elevated likelihoods of experiencing a fresh silent cerebral infarct (41% versus 18%) and anatomical changes in sulci and ventricles associated with old age and cognitive decline [69]. Therefore, the development of new neurological symptoms or further cognitive decline should prompt repeated neuroimaging.

## 5. Effect of Treatment for Atrial Fibrillation on Cognitive Function

Different treatment modalities are used for the management of AF, including rate and rhythm control agents, anticoagulation, electrical cardioversion, and catheter ablation therapy. While the effect of most of these on cognitive function is still unclear, some agents such as anticoagulation have shown a protective role on cognitive function. In this section, we explore the current understanding of the cognitive effects of different treatment modalities for AF and discuss future directions and ongoing trials regarding the same.

### 5.1. Rate and Rhythm Control Strategies

Rate versus rhythm control strategies have been debated over time as to which is superior for combating the progressive cognitive decline that is seen in AF populations. One study directly compared the effect of rate and rhythm strategies on cognition [70]. It involved 1082 patients with AF at a mean age of 80.6 years old who underwent randomization into three different groups that received either rhythm control therapy, rate control therapy, or neither rhythm nor rate control therapy. Cognitive performance measured by the Short Blessed Test suggested that in AF patients not receiving optimal anticoagulation, rhythm control may be protective against cognitive decline over time.

The Early Treatment of Atrial Fibrillation for Stroke Prevention Trial (EAST-AFNET 4) evaluated early rhythm control with either antiarrhythmic agents or catheter ablation in AF populations and also explored cognitive function by measuring the Montreal Cognitive Assessment (MoCA) as a secondary outcome [71]. This study suggested that there might not be a difference in the effect of cognitive function between early rhythm control and usual treatment that comprised initial treatment with rate control and the use of rhythm control agents only for symptom management. However, it is essential to note that this study evaluated cognitive function as a secondary outcome, and the follow-up for this endpoint was only two years. As previously discussed, the computational study completed by Saglietto et al. showed that rates of less than 50 bpm and rates greater than 130 bpm were associated with cerebral hypoperfusion [72]. This underscores the importance of rate control strategies that aim to keep heart rate between 50–130 bpm as it leads to less hypoperfusion and, in theory, would lessen the cognitive burden over time.

### 5.2. Anticoagulation

Several research studies and clinical trials have examined the role of different anticoagulants on the development of cognitive decline in AF populations. Many such studies have suggested that anticoagulants protect against cognitive decline. A retrospective registry study conducted in Sweden showed a reduction in the risk for dementia for patients taking both NOACs and warfarin [73]. An observational study by Madhavan et al. showed a reduction in the risk of dementia with warfarin therapy. It also showed that an increased time in the therapeutic range of warfarin is associated with a reduced risk of dementia [74]. Similarly, another retrospective study by Jacobs et al. also demonstrated time outside of the therapeutic range as being associated with long-term risk of dementia [75]. Therefore, it is important to consider if anticoagulation should be considered in low-risk AF patients who otherwise do not have an indication for anticoagulation. As discussed later, the Blinded Randomized Trial of Anticoagulation to Prevent Ischemic Stroke and Neurocognitive Impairment in AF (BRAIN-AF trial; ClinicalTrials.gov ID: NCT02387229) is ongoing to assess the efficacy of rivaroxaban in reducing cognitive decline in AF patients with a low CHA_2_DS_2_VASc score.

As previously discussed, it is widely regarded that the increase in microbleeds is associated with cognitive impairment [62]. Anticoagulation increases the risk of developing new cerebral microbleeds, as demonstrated in a prospective study by Zhuang et al. comparing non-vitamin-K oral anticoagulants (NOAC) and warfarin treatments [76]. This study evaluated 72 patients, whereby 29 were assigned to a NOAC group, and the other 43 were assigned to a warfarin group. They were followed over one year and monitored for the development of microbleeds using susceptibility-weighted imaging. They found that the rate of development of microbleeds in the NOACs group was 3.4%, compared to the warfarin group, where the rate of development of microbleeds was 20.9%. This study highlights that microbleeds can be significantly reduced when using NOACs rather than warfarin, theoretically decreasing the risk of cognitive impairment.

On the other hand, the Cognitive Impairment Related to Atrial Fibrillation Prevention Trial (GIRAF) was a randomized controlled trial that compared cognitive endpoints in patients with AF receiving either warfarin or dabigatran [77]. The GIRAF study compared dabigatran and warfarin over two years and measured differences in the MoCA, Mini-Mental Status Exam (MMSE), and a variety of other tests assessing cognition. They concluded that there was no statistically significant difference in cognitive endpoints after two years. The Impact of Anticoagulation Therapy on the Cognitive Decline and Dementia in Patients With Non-Valvular Atrial Fibrillation (CAF) trial is another randomized control trial that similarly compared the impact of dabigatran and warfarin therapy on the cognitive function of AF patients [78]. They found that the use of dabigatran and warfarin showed similar rates of stroke, dementia, and cognitive decline at the two-year mark. The equivalent clinical outcomes of NOACs and warfarin despite an increased risk of microbleeds observed in NOACs suggest that the mechanism through which anticoagulants protect against cognitive decline is through the prevention of embolic events despite an elevated risk of microbleeds.

### 5.3. Catheter Ablation

Catheter ablation is known to be associated with peri-procedural ischemic events; however, the effect of catheter ablation on neurocognitive function is still undefined. In an observational study completed by Hsieh et al., 787 patients with AF were divided into two cohorts and followed over nine years [79]. They concluded that at a nine-year follow-up, the group that received catheter ablation had a lower incidence of dementia than the group that did not receive ablation therapy. This effect was more pronounced in patients with AF over 65 years old. On the other hand, another study that included 60 patients with paroxysmal AF and 30 patients with persistent AF undergoing catheter ablation demonstrated a higher prevalence of post-operative neurocognitive dysfunction in both groups at both day 2 and day 90 of follow-up [80].

A pilot longitudinal study completed by Tatewaki et al. examined whether AF ablation would alter regional cerebral blood flow and the brain microstructure [81]. After a follow-up period of six months, it was found that post-cardiac-ablation-therapy, there was an improvement in regional cerebral blood flow in the left posterior cingulate cortex and a decrease in the cortical thickness of the posterior insular cortex. The posterior cingulate cortex is a major component of the limbic system, while the insular cortex is primarily responsible for an array of executive and autonomic functions, including autonomic, gustatory, auditory, and speech and language functions [82]. The authors thus hypothesized the positive impact of catheter ablation on cognitive dysfunction in AF patients.

A randomized control trial completed by Al-Kaisey et al. evaluated the effect of cardiac ablation in AF on cognition [83]. This prospective study randomized 96 AF patients with prior failure of antiarrhythmic drug therapy into two groups—continuing medical therapy versus ablation therapy. In this study, early post-operative cognitive dysfunction was observed in the catheter ablation group, but this effect was transient, and these patients had recovered by the end of the 12-month follow-up period. This study also demonstrated improvement in overall cognitive functioning scores in the group of 49 patients who underwent catheter ablation compared to the group that received medical treatment. This group also had a greater reduction in AF burden and decreased use of antiarrhythmic drugs compared to the medication group and hence this improvement in cognitive functioning is postulated to be due to improved hemodynamic control and the cessation of antiarrhythmic drugs. A large nationwide database study compared dementia risk among AF patients receiving catheter ablation or medical therapy. It was observed that at a median follow-up of 52 months and after adjusting for stroke and other confounding factors, catheter ablation was associated with a decreased dementia risk compared to medical therapy [84].

Overall, the current literature suggests that catheter ablation can benefit cognition in the long term. However, more research is ongoing in this area. As discussed later, two ongoing clinical trials, the Cognitive Impairment in Atrial Fibrillation study (DIAL-F; ClinicalTrials.gov ID: NCT01816308) and the Neurocognition and Greater Maintenance of Sinus Rhythm in Atrial Fibrillation (NOGGIN-AF) trials, will provide more future insight into the effect of catheter ablation compared with medical management in AF patients and subsequent cognitive effects.

### 5.4. Cardioversion

A prospective study by Kedžo et al. studied the role of electric cardioversion on cognition in 25 patients with persistent AF [85]. Cognitive outcomes were assessed based on patient-reported information and the study demonstrated no difference in cognitive function before and after electrical cardioversion. Another study by Bellman et al. involved 50 patients with persistent AF that underwent electrical cardioversion and the MoCA score was used to compare cognitive function before and at a mean of 2 weeks after cardioversion [86]. The study revealed that there was not a significant difference in cognition before and after cardioversion. In a study conducted in Iceland, cerebral blood flow was measured using MRI for AF patients who underwent successful cardioversion followed by maintenance of sinus rhythm [87]. An improvement in cerebral blood flow was observed in the group of patients who had successful cardioversion, whereas such an improvement was not seen in the group who were not successfully cardioverted. The ongoing trial The Norwegian Atrial Fibrillation and Stroke Study (NOR-FIB2, ClinicalTrials.gov ID: NCT03816865) is going to assess for silent cerebral infarcts and their likely etiologies by using diffusion-weighted sequences in MRI in patients with AF undergoing electrical cardioversion.

## 6. Clinical and Socioeconomic Aspects of Association

As per the results published in the Global Burden of Disease, Injuries, and Risk Factors Study, 2019, over the 30-year study duration (1990–2019), there has been a 41% rise in the crude incidence of AF and a 71% rise in the crude incidence of dementia. Consequently, in those 30 years, the crude mortality rate doubled for both diseases individually, indicating a steadily mounting socioeconomic burden associated with them [2,88]. In light of the recently acknowledged independent connection between AF and cognitive decline, it is important to consider cognitive screening in this population [89]. With the widespread availability of basic diagnostics, telemedicine, wearable diagnostic devices, and affordable tools like automated pressure measurement for detecting arrhythmias, neglecting efforts in AF and dementia prevention is no longer acceptable [90]. If early signs are detected, interventions should be directed to prevent or delay the progression via risk identification modification, regular monitoring, patient education, identifying patient-specific outcome priorities, and building an evidence-based treatment strategy aimed at interrupting the detrimental causal link and reducing the proportion of cognitive decline attributable to AF. Furthermore, upon diagnosing AF, it is advisable to promptly establish a baseline cognitive function profile through screening.

It is crucial to understand at this stage that cognitive decline in AF patients often co-occurs with a huge burden of psychosocial impairment including depression and anxiety. In a prospective study conducted on 218 symptomatic AF patients, impairment prevalence rates in three domains—depression, anxiety, and cognition—were studied. Compared to controls without cognitive or psychosocial impairment, participants with impairments in two or three domains showed significantly poorer atrial-fibrillation-specific quality of life at the 6-month assessment. Such patients tended to be older, belonged to lower socioeconomic strata, had a positive smoking history, and had poor educational qualifications [91]. AF patients with cognitive decline also demonstrate inferior adherence to anticoagulation, greater probability of experiencing adverse cardiovascular events, and worse functional decline when contrasted with AF patients without cognitive decline [92]. In conclusion, future research on AF and cognitive decline must incorporate the impacts of social determinants of health on the outcomes associated with it [93].

## 7. Cerebral Amyloid Angiopathy and AF

Cerebral amyloid angiopathy (CAA) is caused by the deposition of B-amyloid in blood vessels [94]. This leads to weakening of the vessel wall and can result in blood vessel rupture which can lead to intracerebral hemorrhage, in turn resulting in cognitive impairment. The Boston Criteria have been validated for the diagnosis of CAA and classify individuals into four main categories for whom CAA is suspected: definite, probable with supporting pathology, probable CAA, and possible CAA [95]. It is important to note that for a definite diagnosis of CAA, pathological evidence through biopsy or autopsy is required. Probable and possible CAA relies heavily on radiological evidence primarily through MRI or CT imaging with an age greater than 55 years being a part of the diagnostic criteria.

It has previously been established that antithrombotic agents lead to an increase in the number of disabling intracranial hemorrhages in CAA patients [96]. One of the mainstays of AF treatment includes anticoagulation; thus, a conundrum arises when patients have both AF as well as CAA [97]. Current evidence suggests anticoagulation in AF should be avoided in the case of patients that have probable or confirmed CAA and lobar intracerebral hemorrhage [94]. Therefore, it is important to consider the burden of hemorrhage and the risk of a thromboembolic phenomenon on a case-by-case basis. Additionally, more randomized control trials are required to further characterize the relationship between AF and CAA and provide management recommendations in patients that have both AF and CAA.

## 8. Discussion

It has been established that AF is associated with cognitive impairment, irrespective of a history of stroke or other shared risk factors. Some studies have shown the association between AF and dementia to be more significant in younger individuals compared to older individuals. The fact that the association is weaker in older individuals who otherwise have more shared risk factors also points to the association being less likely due to common risk factors alone. The current literature has inconsistencies in the role of gender in the association between AF and cognitive impairment and is an area of research that needs to be further explored. Different pathophysiological mechanisms have been proposed to describe this association, including silent cerebral infarcts, cerebral microbleeds, cerebral hypoperfusion, and inflammation and atherosclerosis. A combination of these mechanisms may likely play a role in this association. Neuroimaging can serve as a potential tool in altering treatment decisions including the need for anticoagulation, but more studies are needed on this topic. Assessing the efficacy of MRI-detected silent cerebral infarcts in enhancing risk assessment for future stroke and cognitive dysfunction; evaluating the prognostic significance of cerebral microbleeds’ presence, distribution, and burden for future intracerebral hemorrhage; and exploring the applicability of MRI findings in guiding therapeutic decisions, such as the selection between vitamin K antagonists and DOACs for individual patients, are prospective avenues for neuroimaging research aimed at tailoring AF treatment. The effect of rate and rhythm control agents as well as that of electrical cardioversion on cognitive outcomes of AF patients remains uncertain. Anticoagulants, both warfarin and NOACs, have shown efficacy in reducing cognitive decline in AF patients, and further studies are ongoing to assess the safety and efficacy of oral anticoagulants in AF patients with a low CHA_2_DS_2_-VASc score. While catheter ablation is associated with an increased risk of post-operative cognitive dysfunction in the short term, studies have shown an overall decreased risk of cognitive dysfunction in the long term in patients after catheter ablation. More research is ongoing in this area, as described in the following paragraph.

## 9. Future Directions and Conclusions

Numerous trials are ongoing to fill in the knowledge gaps that exist regarding the association between AF and cognitive impairments. The Cognitive Impairment in Atrial Fibrillation study (DIAL-F; ClinicalTrials.gov ID: NCT01816308) is a currently ongoing case-control study comparing the incidence of cognitive decline using MoCA scores in AF patients receiving either cardiac catheter ablation or antiarrhythmic drugs. Additionally, the Neurocognition and Greater Maintenance of Sinus Rhythm in Atrial Fibrillation (NOGGIN-AF) trial is another ongoing trial designed to also compare cognitive function and brain structural changes in patients receiving catheter ablation versus standard medical management. The culmination of these two active studies will provide more insight into the effect of catheter ablation on cognitive function in AF patients. Finally, as discussed earlier, the Blinded Randomized Trial of Anticoagulation to Prevent Ischemic Stroke and Neurocognitive Impairment in AF (BRAIN-AF trial; ClinicalTrials.gov ID: NCT02387229) is ongoing to assess the efficacy and safety of rivaroxaban in reducing cognitive decline in low-risk AF patients. It seeks to compare the MoCA and MMSE scores of patients receiving rivaroxaban or placebo therapy in non-valvular AF patients with a low CHA_2_DS_2_VASc score. This trial is expected to allow providers to make more informed decisions regarding anticoagulation in low-risk AF patients. The Screening for Atrial Fibrillation with ECG to Reduce Stroke—a randomized controlled trial (UK-SAFER; trial registration number: ISRCTN72104369) is an ongoing randomized control trial in the UK that will include over 120,000 patients who will be randomized to a control group or screening group. Cognition will be studied as a secondary outcome.

## Data Availability

No new data were created or analyzed in this study. Data sharing is not applicable to this article.
